# Effect of Sociality and Season on Gray Wolf (*Canis lupus*) Foraging Behavior: Implications for Estimating Summer Kill Rate

**DOI:** 10.1371/journal.pone.0017332

**Published:** 2011-03-01

**Authors:** Matthew C. Metz, John A. Vucetich, Douglas W. Smith, Daniel R. Stahler, Rolf O. Peterson

**Affiliations:** 1 School of Forest Resources and Environmental Science, Michigan Technological University, Houghton, Michigan, United States of America; 2 Yellowstone Wolf Project, Yellowstone Center for Resources, Yellowstone National Park, Wyoming, United States of America; University of California, Berkeley, United States of America

## Abstract

**Background:**

Understanding how kill rates vary among seasons is required to understand predation by vertebrate species living in temperate climates. Unfortunately, kill rates are only rarely estimated during summer.

**Methodology/Principal Findings:**

For several wolf packs in Yellowstone National Park, we used pairs of collared wolves living in the same pack and the double-count method to estimate the probability of attendance (PA) for an individual wolf at a carcass. PA quantifies an important aspect of social foraging behavior (i.e., the cohesiveness of foraging). We used PA to estimate summer kill rates for packs containing GPS-collared wolves between 2004 and 2009. Estimated rates of daily prey acquisition (edible biomass per wolf) decreased from 8.4±0.9 kg (mean ± SE) in May to 4.1±0.4 kg in July. Failure to account for PA would have resulted in underestimating kill rate by 32%. PA was 0.72±0.05 for large ungulate prey and 0.46±0.04 for small ungulate prey. To assess seasonal differences in social foraging behavior, we also evaluated PA during winter for VHF-collared wolves between 1997 and 2009. During winter, PA was 0.95±0.01. PA was not influenced by prey size but was influenced by wolf age and pack size.

**Conclusions/Significance:**

Our results demonstrate that seasonal patterns in the foraging behavior of social carnivores have important implications for understanding their social behavior and estimating kill rates. Synthesizing our findings with previous insights suggests that there is important seasonal variation in how and why social carnivores live in groups. Our findings are also important for applications of GPS collars to estimate kill rates. Specifically, because the factors affecting the PA of social carnivores likely differ between seasons, kill rates estimated through GPS collars should account for seasonal differences in social foraging behavior.

## Introduction

Per capita kill rate, the number of kills made per predator per unit time, is one of the most basic statistics for understanding the nature of predation [e.g.], [ 1,2]. Among large mammalian predators, wolves (*Canis lupus*) are the species for which the most is known regarding the causes and consequences of kill rates [e.g.], [ 3]–[5]. Like wolves, many of these predators live in seasonal environments and feed on prey that reproduce once per year. For these large mammalian predators, an adequate understanding of predation requires knowing how kill rates vary throughout the year. In many African systems, the assessment of kill rates throughout the year is possible through continuous visual observations [e.g.], [ 6]–[8]. However, in temperate climates, most empirical assessments of kill rate for terrestrial predators rely on detecting kills on snow-covered landscapes [9], [10], where predator kill sites are more easily detected. Because of these challenges, summer kill rates have been estimated for only a few of these predator-prey systems (e.g., [5], [11], [12]).

Monitoring predators with GPS collars is an increasingly common means of estimating the kill rates of large, terrestrial carnivores [e.g.], [ 5], [12,13]. This method involves detecting predation events by searching spatially-clustered locations where predators had recently been [13], [14]. For wolves, per capita kill rate is (to our knowledge always) calculated as the number (or biomass) of prey killed by a pack, divided by pack size, and then divided by the duration for which the observations were made [10]. For this reason, it may seem appropriate to estimate the kill rate for a pack from the kill sites detected through a single GPS-collared wolf. However, special consideration may be required for wolves because all individuals belonging to the group do not always forage together. Specifically, packs are less cohesive as pack size increases [3] and during the summer [15]. Therefore, there is good reason to think that a single monitored wolf would not detect all of the kills made by a pack, leading to underestimates of per capita kill rate. Similar considerations would also be necessary for utilizing GPS collars to estimate the kill rates of other social carnivores (e.g., African wild dogs [*Lycaon pictus*], African lions [*Panthera leo*], spotted hyaenas [*Crocuta crocuta*]), as group cohesion may be influenced by such factors as the presence of young [16], group size [17], and prey size and abundance [18].

A critical reason for estimating kill rate is to assess the extent to which a species' metabolic demands have been met. Of the few studies that have investigated summer kill rates for wolves [3], [5], [19], only Sand et al. [5] attempted to account for the smaller mass of pups by calculating kill rate as kg of prey per kg of wolves living in the pack. What remains unassessed is a simple comparison of how much estimates differ depending on whether or how pups' lower metabolic rates are taken into account [20]. This simple comparison is also useful for understanding how the per capita kill rate estimates for any carnivore species, and in particular those that are social, may be affected by the presence of young.

The primary objective of this study was to estimate how social cohesion affects estimates of summer kill rates for wolves living in Yellowstone National Park, United States. We quantified social cohesion as the probability of an individual wolf attending a carcass fed upon by its pack. To estimate the probability of attendance and summer kill rates, we used principles of the double-count method [21] and pairs of GPS-collared wolves, where each pair lived in the same pack. We compared our estimates of summer kill rates depending on whether or how pups' lower metabolic rates were taken into account. We also used principles of the double-count method to estimate the probability of attendance during the winter by using pairs of VHF-collared wolves. Comparing the probability of attendance between summer and winter allows for a better understanding of the seasonal variation in wolves' social foraging behavior.

## Materials and Methods

### Ethics statement

The handling of all wolves was carried out in strict accordance with approved veterinarian and National Park Service protocols for safe animal welfare and handling. The handling of wolves was also approved by the Institutional Animal Care and Use Committee at Michigan Technological University (IACUC #L0141).

### Study system

**Figure 1:**
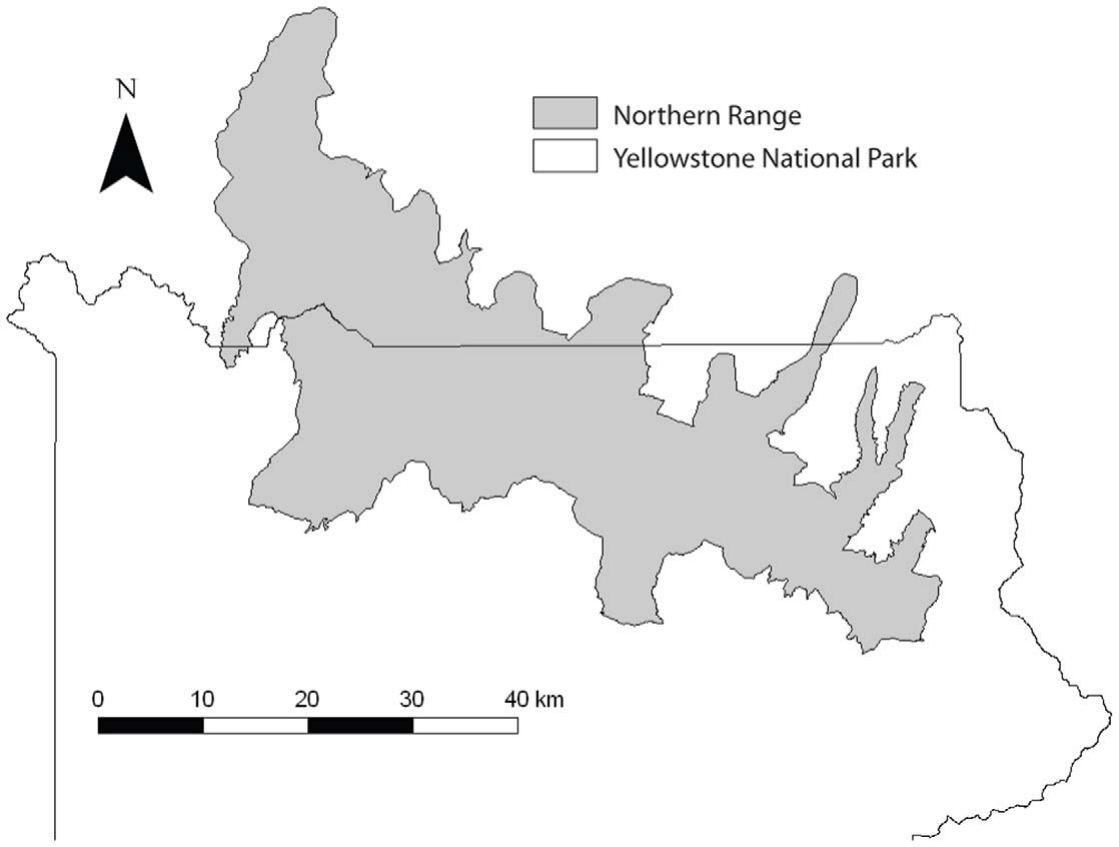
Map of the Northern Range. Northern Range wolves were monitored from 1997 to 2009.

We studied ten wolf packs between 1997 and 2009 (Table 1). These packs lived on the Northern Range of Yellowstone National Park. The Northern Range (1000 km^2^ within Yellowstone National Park) is located in the central portion of the North American Rocky Mountains, and its boundaries are defined by the seasonal movements of the northern Yellowstone elk (*Cervus elaphus*) herd (Fig. 1). Elevations vary primarily from 1,500–2,400 m, with lower elevations characterized by large open valleys of grass meadows and shrub steppe vegetation. Higher elevations are characterized by coniferous forests [22]. The Northern Range is also inhabited by other ungulate species on which wolves occasionally prey [23]. These species are bighorn sheep (*Ovis canadensis*), bison (*Bison bison*), moose (*Alces alces*), mountain goat (*Oreamnos americanus*), mule deer (*Odocoileus hemionus*), pronghorn (*Antilocapra americana*) and white-tailed deer (*Odocoileus virginianus*). Several species of predators are also common, including cougars (*Puma concolor*), coyotes (*Canis latrans*), and black (*Ursus americanus*) and grizzly (*Ursus arctos*) bears.

**Table 1:** Years and seasons during which we monitored various packs for prey acquisition rate and probability of attendance (PA).

Pack	1997	1998	1999	2000	2001	2002	2003	2004	2005	2006	2007	2008	2009
Agate											L		
Blacktail												E	L,S
Druid	E	L,E	L,E	L,E	L,E	L,E	L,E	L,E			L,E	L,E	L
Everts													L,S
Geode							L,E	L,S^1^,E	L				
Hellroaring									E	L,E			
Leopold	E	L,E	L,E	L,E	L,E	L,E	L,E	L,E	L,S^1^,E	L,E	L,S^1^,E	L,S	
Oxbow											E	L,S	
Rose	E	L,E	L,E	L,E	L,E	L							
Slough									L,E	L,E			

The seasons that we monitored various packs were late winter (L), summer (S), and early winter (E). We determined PA for two wolves in each pack during every season that a pack was monitored except for summer periods marked as S^1^. During summer periods marked as S^1^, only one GPS-collared wolf was present in the pack. We determined prey acquisition rates during all summer periods that we monitored a pack of wolves.

### Telemetry collars

As part of a long-term research program, several wolves each year since 1995 have been live-captured and fitted with either VHF (Telonics, Inc. Mesa, AZ) or GPS telemetry collars (see [24] for details). We used downloadable GPS collars manufactured by Televilt (Lindesberg, Sweden) and Lotek (Newmarket, ON, Canada).

### Summer observations

We studied one or two wolf packs each summer from 2004 to 2009, except in 2006. In total, we monitored 11 GPS-collared wolves living in five different packs (Table 1). Study periods averaged 74.7 days (±12.0 SE) and were always between 1 May and 31 July. The duration of each study period and the lack of observations in 2006 were attributable to failure of GPS collars.

#### Pack size and litter size

Estimates of per capita kill rate for wolves require knowing the size of the pack. We assumed the number of adults in a pack during the summer was equal to the pack size during the previous March (see below), except for cases when we knew a wolf had died or dispersed. Because survival rates of pups in our study area are often ≤70% during the first seven months of life [25], the number of pups in a pack typically declines throughout summer. Therefore, we estimated the number of surviving pups for each month (May, June, July) through observations of each pack at their homesite. Typically packs were observed at least once per week. With these observations, we fit linear regression models relating Julian day to the number of pups observed for each pack. We excluded observations judged to be underestimates on the basis of subsequent counts when a larger number of pups were observed. From the regression model, we estimated the number of pups living during each month as the number predicted for the 15^th^ day of each month (see Figure S1).

#### GPS collars

We used information downloaded from GPS collars to find carcass sites during the summer. We programmed the first GPS collar that we used (in 2004) to record 40 locations per day from 1 May – 31 July (see Text S1). In every other year, we programmed the collars to record a location every 30 minutes. Each GPS collar provided usable data, on average, for 81.0 days (±7.9 SE, *n* = 11).

#### Cluster identification

We downloaded locations from GPS collars, on average, every 8.1 days (range: 5–14 days). Following each download, we used either ArcView 3.2 or ArcMap 9.3 (Environmental Systems Research Institute, Redlands, CA, USA) to identify clusters of GPS locations belonging to an individual wolf. We defined a cluster as a set of ≥2 locations where every location in the set is within 100 m of its nearest neighbor.

#### Cluster searches

We hiked approximately 6400 km to search 94.2% of the 1848 clusters. We examined these clusters, on average, 15.6 days (±0.4 SE) after the time when wolves had left the area. Some clusters (5.2%) were near homesites and were never searched. However, these clusters likely did not represent sites where prey were killed. This is because these clusters were associated with wolves travelling repeatedly to and from the homesite, easily distinguishable from non-consecutive locations. A few clusters (0.7%) were far from homesites but could not be searched due to logistical constraints.

Field crews of 2–4 people searched clusters for the remains of ungulate carcasses that had been fed on by members of the pack to which the GPS-collared wolf belonged. Specifically, we searched the ground within 400 m^2^ of each individual location by walking a grid-like pattern of transect lines with the individual location at the center of the 20 m×20 m area. If we detected a carcass, we continued to search the remaining individual locations in order to investigate the possibility of multiple carcasses. Within each cluster, we also searched any other nearby areas where we noticed wolf sign (e.g., bed site). We searched each cluster for, on average, 11.2 minutes (±0.2 SE). Search time was dependent upon the number of people searching, the number and spread of the individual locations, and the vegetative characteristics of the site. For each carcass we discovered, we judged whether the prey had died at about the time wolves had created the cluster. For prey judged to have died at that time, we estimated the date and time of death based on the time the wolf first appeared within 100 m of the carcass site. We estimated carcass biomass for deer and elk through sex and age-specific growth curves, specific to season, developed for our study area [26]. For bison, we used sex and age-specific estimates from our study area [27, Yellowstone National Park, *unpublished data*]. For other ungulate species, we used published weight estimates specific to species, sex, and age class (i.e., calf or adult) [28]. Following Wilmers et al. [29], we estimated edible biomass to be 68% of live weights.

Because wolves often visit carcasses whose edible biomass had been previously consumed [15], we did not count carcasses unless there was evidence that a GPS-collared wolf had consumed significant biomass. Specifically, because small ungulates (≤130 kg [live weight] during the study period) were typically consumed within one day and large ungulates (>130 kg) within three days (see Figure S2), we only counted carcasses where at least two locations occurred within these time periods and within 100 m of the carcass. Because we included all carcasses that provided significant biomass to wolves (i.e., including those scavenged), we estimated and refer to rates of prey acquisition, rather than kill rates.

#### Single, isolated locations

Previous work indicates that remains of prey, especially small non-ungulate prey, are occasionally found at single locations [5]. To assess this possibility in our study system, we searched 1045 single locations in 2008. We rarely found evidence of wolves feeding on carcasses at single locations and did not include these carcasses because wolves did not obtain significant biomass (see Text S2).

### Winter observations

We studied ten wolf packs during 30-day study periods which occurred every early winter (from mid-November to mid-December) and every late winter (March) from November 1997 to March 2009. During each 30-day study period, we observed two or three of these ten packs (Table 1). Each observed pack included at least two VHF-collared wolves. From these observations, we determined the presence or absence of each VHF-collared wolf at ungulate carcasses belonging to that wolf's pack. We assumed all individuals traveling with the pack at first light, and near a fresh carcass, had also been present at the carcass during the night.

These observations were made for 109 individual wolves, some of which were alive and monitored during more than one study period (Table S1). We observed the monitored wolves on a nearly daily basis from either light, fixed-wing aircraft or ground-based observation points. From these observations, we also determined the size of each pack for each study period. For further details, see Smith et al. [23]. From these observations, we detected 852 carcasses where wolf presence could be determined.

We used VHF-collared wolves during the winter because most packs did not contain GPS-collared wolves. We did not use VHF-collared wolves to document presence or absence at carcasses during summer because our field methods did not allow an opportunity to monitor VHF-collared wolves intensively, like had been done during winter.

### Analysis

#### GPS collar success simulations

The eight GPS collars deployed in 2008 and 2009 successfully recorded 98.7% of the programmed locations during download intervals unaffected by the denning behavior of a breeding female (*n* = 2). We successfully downloaded all locations for those years. Success rates were lower for GPS collars during previous summers because not all locations were successfully received during downloads (see below). To assess how the number of carcasses detected by an individual wolf would be affected by reduced success rates, we simulated the effect of reduced success rates by randomly removing a specified proportion of locations for the data collected during 2008 and 2009. We conducted simulations in R version 2.8.1, using methods similar to those of Knopff et al. [30]. More specifically, we simulated 1000 replicate sets of data for each of the eight wolves at several rates of success (30%, 35%, … 95%). For each level of success rate, we calculated the proportion of instances that the GPS locations for individual wolves would have still detected each carcass (i.e., met our spatial and temporal requirements) with the reduced data. We then calculated the mean proportion of carcasses still detected for both large and small ungulates at each level of success rate. We divided these proportions by 0.987 to estimate the proportion of carcasses that would have been detected for a GPS collar with 100% success.

#### Summer prey acquisition rates

Per capita rate of prey acquisition is calculated as the total number (or biomass) of carcasses fed upon by a pack, divided by pack size, and then divided by the time period during which the data was collected. Estimating the number of carcasses is particularly difficult during summer when packs do not forage as cohesively and many prey are smaller. Here we describe how we used principles of the double-count method to estimate rates of prey acquisition for a pack.

According to the double-count method [21], two observers (A and B) attempt to detect the objects being enumerated (commonly individuals in an animal population). The method involves recording the number of objects detected by observer A (*N_A_*), the number detected by observer B (*N_B_*), and the number detected by both A and B (*N_AB_*). From these values, an estimate for the total number of objects, including those undetected by either observer is:
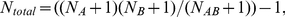
(1)and the probability of detection for an object for observer A and B is: 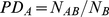
(2a)

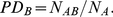
(2b)


We used Eq. 1 to estimate the total number of carcasses during summer for packs that included a pair of GPS-collared wolves. We did this by treating the wolves as observers A and B, and by considering that a wolf detected a carcass if that wolf's GPS locations met the spatial and temporal requirements of carcass detection (See *Cluster Searches*). Estimates of *PD* (Eq. 2) are useful for estimating the number of carcasses acquired by packs that contained only a single GPS-collared wolf. That is, for cases involving a single observer, total abundance may be calculated as [31]: 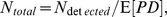
(3)where *N_detected_* is the number of objects detected by the observer and E[*PD*] is the probability of detection expected for that observer. Equation 3 is useful if there is some basis for estimating that observer's *PD*. For several packs that we monitored, only a single wolf was GPS-collared. We estimated rates of prey acquisition for these packs from *N_detected_* (i.e., the number of carcasses detected by the single GPS-collared wolf) and from the mean *PD* for the eight wolves where we had estimated *PD*.

An important assumption of the double-count method is that each object being counted has an equal probability of being detected. In our application, *PD* might vary with respect to prey size. For this reason, we assessed *PD* separately for large and small ungulate carcasses. Large ungulate carcasses consisted primarily of adult elk while small ungulate carcasses included primarily adult deer and neonate ungulates.

#### Effect of low GPS collar success rates

**Figure 2:**
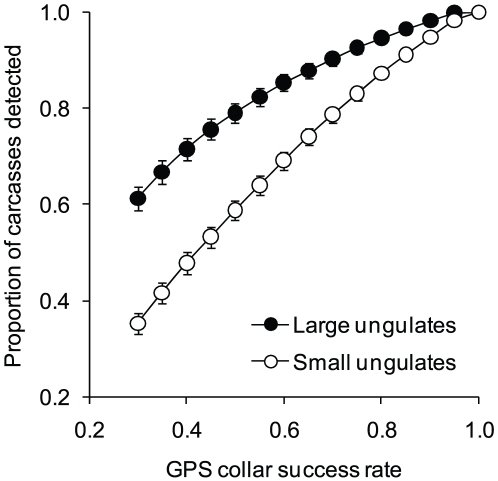
The influence of GPS collar success on carcass detection by individual wolves. Simulated relationship between the proportion of GPS locations successfully acquired and the mean proportion of carcasses still detected (*n* = 183 large ungulate carcasses, *n* = 174 small ungulate carcasses). Error bars represent standard errors, many of which are too small to see. The lines represent best fitting polynomial regressions: *y* = 0.29+1.29*x* – 0.58*x* ^2^ (*R^2^* = 1.00, *P*<0.0001) for large ungulates and *y* = – 0.08+1.59*x* – 0.50*x* ^2^ (*R^2^* = 1.00, *P*<0.0001) for small ungulates.

Instances where packs included only a single GPS-collared wolf also happened to be associated with GPS collars which had relatively low success rates for each download interval (i.e., 73.3%±2.9% [mean ± SE]). Before applying Eq. 3, we adjusted *N_detected_* according to the simulated relationship between collar success rate and the probability of carcass detection (see Fig. 2).

Recall, the value of *N_detected_* for the wolf wearing a GPS collar in summer 2004 represents another special case because of its unique schedule for recording locations. To account for this difference, we adjusted the number of large and small ungulates expected to have been detected (see Text S1).

#### Pups and prey acquisition rates

Calculating pack size for summer periods is complicated by pups being much smaller than adults. Pack size could be calculated in any of four different ways: (*i*) count pups as though they were adults, (*ii*) do not count pups, because they are small and eat little, (*iii*) explicitly account for pups' smaller biomass or, (*iv*) explicitly account for pups' lower metabolic rates. More specifically, one could convert each pup into the number of adult equivalents that a pup represents by these expressions: (*mass_pup_*/*mass_adult_*) to account for biomass or (*mass_pup_^3/4^*/*mass_adult_^3/4^*) to account for metabolic rate. Hereafter, we use the phrase metabolic-rate-adult-equivalent wolves to indicate pack size was determined while using the expression (*mass_pup_^3/4^*/*mass_adult_^3/4^*). We calculated and compared per capita prey acquisition rates using each method for each month of summer (*n_May_* = 6, *n_June_* = 7, *n_July_* = 5). To do so, we assumed *mass_pup_* in May, June, and July was 2.7 kg, 7.5 kg, and 12.2 kg, respectively [32].

We also assumed adult wolves weighed 43.4 kg in May. This is the mean mass of Northern Range wolves when they are weighed in winter (Yellowstone Wolf Project, *unpublished data*). We assumed May weights were similar to winter weights because wolves tend to acquire similar amounts of biomass during these periods [33]. However, wolves tend to acquire less biomass in June and July [33], and wolf mass tends to be less during summer [15]. Therefore, following Peterson et al. [15], we assumed that June and July weights were 92% of winter weights.

We used regression analysis to assess rates of prey acquisition as a function of pack size (metabolic-rate-adult-equivalent wolves). For this analysis, we calculated prey acquisition rate for each instance that a pack was monitored during June and/or July (*n* = 7). Study periods averaged 48.2 days (range: [10.7, 61.0]). We limited this analysis to carcasses fed upon during June and July because the relationship between food availability and pack size is well understood during winter [34], [35] and wolves acquire biomass at rates similar to winter during May [33].

#### Seasonal variation in carcass attendance

The probability of detection (Eq. 2) is also the probability of our detecting that a wolf had been in attendance at a carcass. The probability of attendance (PA) quantifies an important aspect of wolf foraging behavior. That is, PA quantifies the cohesiveness of foraging for individuals within a pack.

We calculated estimates of PA (using Eq. 2) for each of the summer months for each of the eight GPS-collared wolves living in the four packs which each contained two GPS collars (Table 1). From these observations, we recorded the presence or absence of wolves at 141 small and 120 large ungulate carcasses. These observations yielded 46 estimates of PA. Moreover, each estimate could be characterized by several factors (i.e., the individual wolf, pack to which the wolf belonged, reproductive status of the wolf [yes or no], pack size [small or large], year [2008 or 2009], and month [May, June, July]). We used SPSS version 9.0 (SPSS, Inc., Chicago IL) to assess general linear models for the purpose of better understanding how PA might be affected by these variables.

We also used Eq. 2 to estimate PA during the winter for VHF-collared wolves monitored between November 1997 and March 2009 (Table 1). Although many packs have more than two wolves marked with VHF collars, we calculated attendance rates for two randomly selected individuals from each pack for each early-winter and late-winter study period (see Table S1). Because we were interested in the attendance patterns of wolves regularly with the pack, we did not consider radio-collared individuals that dispersed from the pack or individuals that were only rarely observed with the pack during the study period. Through these wolves, we detected 839 carcasses. Of these, we were able to determine the size for 807 (i.e., 555 large ungulates and 252 small ungulates).

For this 12-year period, we calculated PA for small and large ungulate carcasses for each winter study period. These calculations yielded 260 monthly estimates of PA. Moreover, each estimate could be characterized by several factors (i.e., the individual wolf, pack to which the wolf belonged, social status of the wolf [alpha or subordinate], sex, age class [pup, yearling, adult], pack size, prey size [large or small], year [2008 or 2009], and study period [early winter or late winter]). We assessed general linear models to better understand how PA might be affected by these factors.

## Results

### GPS collar success simulations

Our simulations show that the probability of detecting a carcass declines as GPS collar performance declines (Fig. 2). For example, a GPS collar with a simulated success rate of 75% would be expected to detect only 93% of large ungulate carcasses and only 83% of small ungulate carcasses. The proportion of small ungulate carcasses predicted to be detected is less than that for large ungulate carcasses because large ungulate carcasses were associated, on average, with a greater number of GPS locations (9.98±0.70 [mean ± SE]) than were small ungulate carcasses (4.70±0.30 [mean ± SE]; Figure S3).

### Summer prey acquisition rates

**Figure 3:**
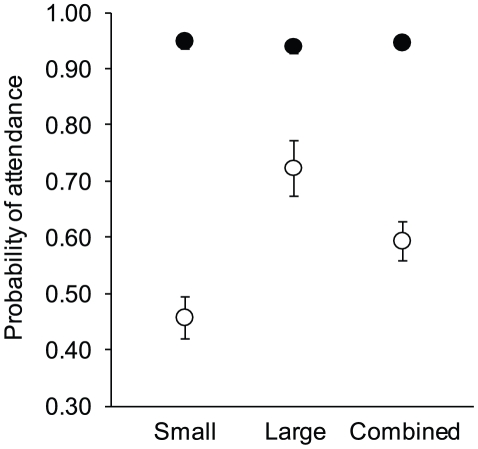
Probability of carcass attendance depending on prey size and season. Points represent the mean probability of attendance for a monitored wolf at the carcass of a small ungulate (≤130 kg), large ungulate (>130 kg), or all ungulate carcasses combined. Open circles represent summer months and filled circles represent winter months. The error bars represent standard errors, some of which are too small to see.

The mean probability of attendance (PA) was 0.72 (±0.05 SE, *n* = 8) for large ungulate carcasses and 0.46 (±0.04 SE, *n* = 8) for small ungulate carcasses. These values differ significantly (*t* = 5.75, *P*<0.001; paired t-test; Fig. 3). These are also the values we used in Eq. 3 to estimate prey acquisition rates for packs with only a single GPS-collared wolf.

If we had ignored the tendency for individual wolves not to visit every carcass fed upon by its pack (i.e., if we had assumed PA equals one), then we would have grossly underestimated daily prey acquisition rates. Specifically, our estimates would have been, on average, only 58% of those that did account for PA when measured as number of ungulates per metabolic-rate-adult-equivalent wolf, and only 68% for those measured as biomass per metabolic-rate-adult-equivalent wolf.

**Figure 4:**
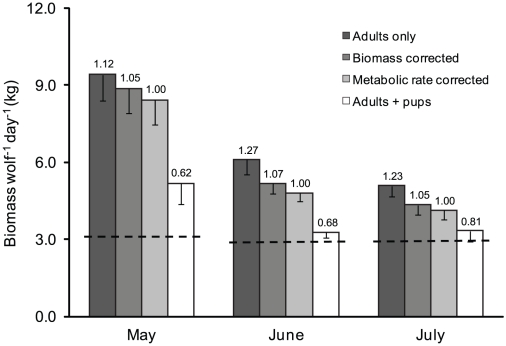
Estimates of per capita rates of prey acquisition based on different methods for determining pack size. The number above each bar represents the rate in proportion to the metabolic corrected rate. Vertical bars represent the standard error. The dashed lines represent the estimated minimum daily energetic requirement (3.84 and 3.59 kg wolf^−1^ day^−1^ in May and June/July, respectively) for adult wolves in our study area [37].

The estimated rates of daily prey acquisition (edible biomass of ungulates per metabolic-rate-adult-equivalent wolf) were 8.4 kg (±0.9 SE; range: [5.6, 11.4]) in May, 4.8 kg (±0.3 SE; range: [3.8, 6.4]) in June, and 4.1 kg (±0.4 SE; range: [3.5, 5.6]) in July. Moreover, estimated rates of prey acquisition varied importantly with various methods for calculating pack size, as estimates varied by as much as 50% depending on how newborn pups were accounted for when determining pack size (Fig. 4).

**Figure 5:**
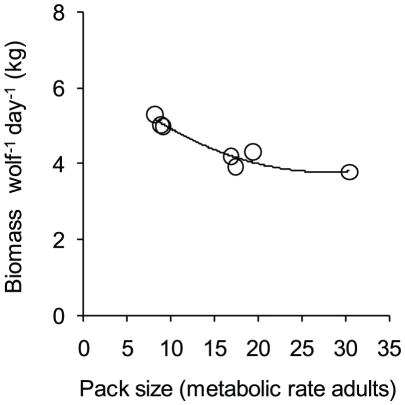
Rates of prey acquisition (kg wolf^−1^ day^−1^) during June and July in relation to pack size. Pack size is determined as the number of metabolic-rate-adult-equivalent wolves (see *Pups and prey acquisition rates*). The line represents the best fitting polynomial regression: *y* = 6.58 – 0.20*x*+0.004*x* ^2^ (*R^2^* = 0.92, *P*<0.01).

The amount of biomass acquired per metabolic-rate-adult-equivalent wolf during June and July declined as pack size increased (Fig. 5). More specifically, a polynomial regression explained 92% of the variation in prey acquisition rates (*R^2^* = 0.92, *P*<0.01). For context, a simple linear regression explained 79% of the variation (*R^2^* = 0.79, *P*<0.01).

### Seasonal variation in carcass attendance

The general linear model for PA that we attempted to fit to the summer data could not calculate the statistical significance of several factors when all factors were included in the model (i.e., wolf, pack, breeding status, pack size, prey size, year, and month). This failure arose in part because the seven factors are supported by only 46 observations and because several factors are correlated. In particular, pack, pack size, and year are correlated; and breeding status, pack size, and wolf are correlated. Because this full model failed, we constructed a reduced model including what we expected to be the most important and ecologically-relevant variables, but at the same time contained fewer pairs of correlated covariates. Specifically, we fit a model that contained four of the seven covariates that had appeared in the full model (i.e., pack size, month, prey size, and breeding status). For this model, pack size (*P* = 0.41), month (*P* = 0.18), and breeding status (*P* = 0.86) were not significant, but prey size did have a significant influence on PA (*P* = 0.004). Considering this to be the full model, we then used backward elimination until we found a model containing only p-values that were <0.05. From this set of models, we selected the most parsimonious (i.e., the model with the lowest AIC_c_). By this process, the most parsimonious model included only prey size (*P* = 0.003).

As had been the case for the summer data, factors like year, pack, and wolf were also correlated for the winter data. Also, these factors were correlated with sex and pack size. To gain at least a tentative understanding of the ecological factors that might affect PA during winter we constructed a general linear model that included what we expected to be the most important and ecologically-relevant variables, but at the same time contained fewer pairs of correlated covariates (i.e., included these factors: sex, age class, social status, study period [early or late winter], prey size, and pack size). This model suggests that age class (*P*<0.001) and pack size (*P* = 0.03) have an important influence on PA during winter, but that social status (*P* = 0.42), study period (*P* = 0.88), prey size (*P* = 0.57), and sex (*P* = 0.27) are not important factors.

**Figure 6:**
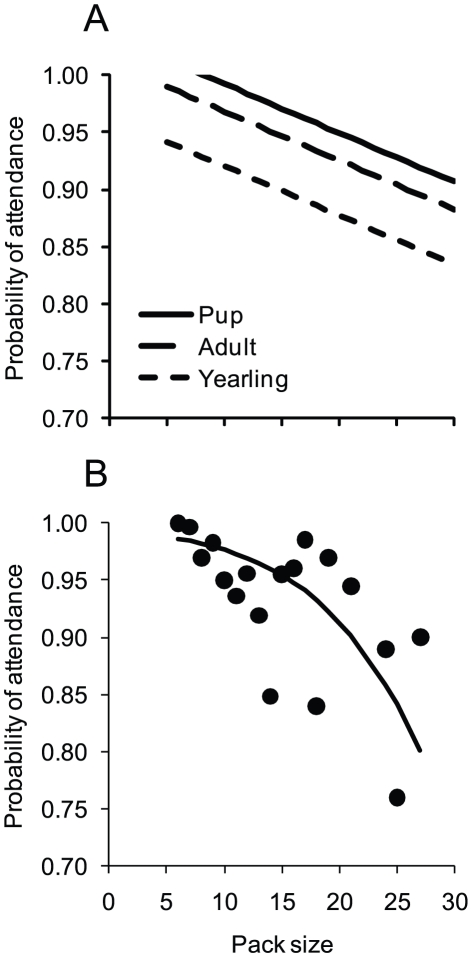
The influence of pack size and wolf age on the probability of carcass attendance during winter. Panel (A) depicts predictions for general linear model that included the influence of pack size and wolf age. For panel (B), the curve represents the linear regression fit to a logit transformation of mean probability of attendance and then back transformed. The regression also weighted each observation according to each observation's sample size. The model is *y* = (1+exp[–{5.10–0.14*x*}])^−1^ with *R^2^* = 0.43 and *P* = 0.003.

Considering this to be the full model, we then used backward elimination until we found a model containing only p-values that were <0.05. From this set of models, we selected the most parsimonious (i.e., the model with the lowest AIC_c_). The most parsimonious model included only age class (*P*<0.001) and pack size (*P*<0.001). This general linear model predicts that PA is greatest for pups and least for yearlings. More specifically, for pack size of 13 (the average pack size for our data), PA is 0.98 for pups, 0.96 for adults, and 0.91 for yearlings (Fig. 6A). Also, PA tends to decline with increasing pack size (Fig. 6B).

Previous work suggests carcass attendance rates should be greater during winter than summer because pack cohesion declines during summer [15]. Accordingly, our data supports the inference that wolves are more likely to attend a carcass during the winter than during summer (PA_winter_ = 0.95±0.01 SE, PA_summer_ = 0.59±0.04 SE; *P*<0.0001, *t* = 9.62, *n_summer_* = 8, *n_winter_* = 140; t-test; Fig. 3).

## Discussion

An underappreciated aspect of social carnivores is how their foraging behavior may vary among seasons. Seasonal variation in the foraging behavior of social carnivores is relevant to our understanding of carnivore sociality and the estimation of kill rates through GPS collars. This is of particular importance to the management and conservation of species because GPS collars are an increasingly common means of estimating kill rates for large, terrestrial carnivores.

Our results show how the accuracy of such estimates depends critically on how details like GPS collar performance (Fig. 2), social behavior (Fig. 3), and pups' metabolic rate (Fig. 4) are taken into account. Our results, interpreted in the context of related efforts [5], [30], indicate that the accuracy of estimates for kill rate derived from GPS collars depends on accounting for details that may be important in some cases, but not others. For example, the accuracy of kill rate estimates appears more sensitive to accounting for collar performance in social carnivores. More specifically, simulations based on data collected from cougars, a solitary carnivore, indicated that a GPS success rate of 45% was associated with still detecting 95% of carcasses [30]. By contrast, our simulations indicated that a collar success rate of 45% would result in detecting only 75% of large ungulate carcasses and only 53% of small ungulate carcasses originally detected (Fig. 2). Although detection rates tend to decline as the frequency of locations declines [14], [36], we collected locations six times as frequently as did Knopff et al. [30]. As suggested by Knopff et al. [30], the difference between these results is likely because the social nature of wolves leads to shorter handling time of carcasses. Moreover, our simulated wolf results were based on data collected during summer when individual wolves often intermittently leave carcasses to return to their homesite.

For social carnivores like wolves, the accuracy of kill rates collected through GPS collars are also likely to depend upon accounting for the cohesiveness of foraging behavior. In order to account for this behavior while estimating summer kill rates, we utilized principles of the double count method [21]. An important assumption of the double count method is that the probability of detection by one observer has no effect on the probability of detection by the other observer (i.e., the observers are independent). Although the evaluation of this assumption is likely not possible for our application of the double count method, the most likely situation is that the detection of a carcass by one wolf is associated with an increased chance that the other wolf would also detect that carcass. This situation would have the effect of deflating *N_A_* and *N_B_* and inflating *N_AB_*, which in turn would cause *N_total_* (see Eq. 1) to underestimate the total number of carcasses. While this underestimation is undesirable, it is important to keep in mind that this method produced estimates of summer prey acquisition rates that were, on average, 85% (number of prey) and 62% (biomass of prey) greater than estimates based on data obtained through a single GPS-collared wolf. The best perspective may be to appreciate traditional methods of estimating kill rate assume that each wolf attends every carcass. The method we used, while it likely violates the assumption of independence, is still an important improvement over previous methods which presume that each wolf attends every carcass.

Our results also provide a sense of how the influence of sociality, and its tendency to vary with prey size, age of the predator, and group size, is likely to differ between seasons (Figs. 3, 6). More specifically, for wolves, PA is influenced by prey size only during summer (Fig. 3). However, PA is not uniform during winter and is best explained by the age of the wolf and the size of its pack (Fig. 6). It is reasonable to hypothesize that a larger data set would show that PA also varies with other ecological factors like prey availability, predator density, and climatic conditions. Moreover, similar factors also influence the foraging behavior of other social carnivores. For example, among spotted hyaena clans, individuals are more likely to be found with other clan members during periods when migratory prey are available. Additionally, the number of individuals present at a carcass tends to increase as prey size increases [18]. As such, prey abundance and size would be likely to influence PA for hyaenas. An increased understanding of the factors that influence PA for social carnivores is critical for determining the nature of predation, and how it varies between species and study area.

Because GPS collars allow for consistent detection of carcasses during snow-free periods of time, the estimation of summer kill rates, in particular, is becoming increasingly common [e.g.], [ 5,12]. For our study area, this is also the time period associated with the growth of newborn wolf pups. Our work highlights that the most useful estimates of per capita kill rates, during such periods of reproduction, should account for the reduced metabolic requirements of young (Fig. 4). This is especially true if a primary purpose of estimating kill rate is to understand how much food each predator is acquiring.

Previous work suggests that the amount of biomass of food available per wolf declines as pack size increases, both during winter [34], [35] and summer [5]. Our work also shows that the amount of food available per individual (when correcting pack size for differences in metabolic rates) decreases as pack size increases (Fig. 5). Further, our results suggest that, on average, wolves obtain biomass in excess of their minimum daily energetic requirements during summer (Fig. 4). Specifically, wolves acquired ∼4.5 kg/wolf/day during June and July, which is 25% greater than the biomass needed to meet their energetic requirements (3.6 kg/wolf/day) [37]. Nevertheless, wolves lose weight during summer [15]. A possible explanation for this is that wolves often lose much of what they acquire to scavengers [29]. Although avian scavenging declines during the summer (Yellowstone Wolf Project, *unpublished data*), we found evidence of bears scavenging carcasses at about half of all large ungulate carcasses during June and July (Yellowstone Wolf Project, *unpublished data*). Because grizzly bears often usurp significant biomass when scavenging [38], Yellowstone wolves likely consume biomass much closer to their minimum requirements. These considerations further suggest that the loss of biomass to scavengers is an underappreciated aspect of foraging ecology [39].

Our findings also draw attention to the dynamic nature of group cohesiveness (measured as PA) as a basic feature of group living (Figs. 3, 6). Assessment of how and why animals live in groups has largely focused on explaining group size [e.g., 39]. However, our work suggests that adequate explanations of sociality may require accounting not only for group size, but also group cohesiveness. Pack cohesiveness may have underappreciated fitness consequences for its members. For example, the perceived decline in per capita kill rate with increasing pack size [e.g., 39] may be at least a partial artifact of not accounting for the tendency of larger packs to forage less cohesively. That is, core members' intake rates may not decline with increasing pack size, if increasing pack size also means that the entire group tends not to be present at every carcass.

## Supporting Information

Figure S1 Linear models of pup survival for the seven packs for which summer prey acquisition rates were estimated. The y-axis displays high counts of pups observed throughout the summer monitoring period, beginning 1 May. Each panel (A-G) displays the equations from which pup survival was determined, with dates for which each equation was used in parentheses. Note the different *x* - and *y*-axis scales. The table located in the top-right of Fig. S1 displays the number of pups born and the predicted number of pups surviving for each pack on 15 May, 15 June, and 15 July.
(EPS)

Figure S2 Proportion of detected carcasses for which the number of individual wolf locations within the 100 m carcass buffer no longer increased following the time period. For small ungulates (*n* = 174), >85% of carcasses are no longer active after 1 day while >85% of large ungulate carcasses (*n* = 183) are no longer active after 3 days.
(EPS)

Figure S3 Frequency of observations for the number of individual wolf locations within 100 m of a carcass for large (*n* = 183) and small (*n* = 174) ungulates during the allowed time period for carcass detection (i.e., 1 day for small ungulates, 3 days for large ungulates).
(EPS)

Table S1 Pack affiliation for individual wolves during winter monitoring periods, 1997–2009.
(DOC)

Text S1 Description of the GPS collar used during summer 2004.
(DOC)

Text S2 Investigation of single, isolated GPS locations.
(DOC)
