# Supplementary material for: Effect of Sociality and Season on Gray Wolf (Canis lupus) Foraging Behavior: Implications for Estimating Summer Kill Rate
Source: PLoS One. 2011 Mar 1;6(3):e17332. doi: 10.1371/journal.pone.0017332 (PMC3046980; doi:10.1371/journal.pone.0017332)
Supplement: Table S1 — Pack affiliation for individual wolves during winter monitoring periods, 1997–2009. (DOC) [file pone.0017332.s004.doc]

**Table S1. Pack affiliation for individual wolves during winter monitoring periods, 1997-2009.** The monitoring periods were early winter (E) and late winter (L). To be included, the wolf must have been marked with a functioning radio collar and belonged to a pack for which probability of attendance (PA) was determined during the monitoring period (see Table 1). “X” indicates the wolf was a randomly selected individual for which PA was calculated during the monitoring period, while “O” indicates the wolf was present in the pack but did not have a PA calculated during the monitoring period. Wolves in italics are those which belonged to more than one study pack during the 12 winters for which we determined PA.

|  |  | **97-98** | | **98-99** | | **99-00** | | **00-01** | | **01-02** | | **02-03** | | **03-04** | | **04-05** | | **05-06** | | **06-07** | | **07-08** | | **08-09** | |
| --- | --- | --- | --- | --- | --- | --- | --- | --- | --- | --- | --- | --- | --- | --- | --- | --- | --- | --- | --- | --- | --- | --- | --- | --- | --- |
| **Wolf** | **Pack** | **E** | **L** | **E** | **L** | **E** | **L** | **E** | **L** | **E** | **L** | **E** | **L** | **E** | **L** | **E** | **L** | **E** | **L** | **E** | **L** | **E** | **L** | **E** | **L** |
| 2M | Leopold | X | X | X | X | X | O | X | X | O | O |  |  |  |  |  |  |  |  |  |  |  |  |  |  |
| 7F | Leopold | X | X | X | O | O | X | O | O | X | X |  |  |  |  |  |  |  |  |  |  |  |  |  |  |
| 8M | Rose | X | O | O | O | X | X |  |  |  |  |  |  |  |  |  |  |  |  |  |  |  |  |  |  |
| 9F | Rose | X | O | O | O |  |  |  |  |  |  |  |  |  |  |  |  |  |  |  |  |  |  |  |  |
| 21M | Druid |  | O | O | O | X | O | O | O | X | O | O | X |  |  |  |  |  |  |  |  |  |  |  |  |
| 40F | Druid | X | O | O |  |  | X |  |  |  |  |  |  |  |  |  |  |  |  |  |  |  |  |  |  |
| 42F | Druid | X |  | X | O | O | X | X | O | O | X | X | X | O |  |  |  |  |  |  |  |  |  |  |  |
| 56M | Leopold |  | O |  |  |  |  |  |  |  |  |  |  |  |  |  |  |  |  |  |  |  |  |  |  |
| 77F | Rose |  | X | X | O | X | O |  |  |  |  |  |  |  |  |  |  |  |  |  |  |  |  |  |  |
| 78F | Rose |  | O | X | X |  |  |  |  |  |  |  |  |  |  |  |  |  |  |  |  |  |  |  |  |
| 82M | Rose |  | X | O | O |  |  |  |  |  |  |  |  |  |  |  |  |  |  |  |  |  |  |  |  |
| 83M | Rose |  | O |  |  |  |  |  |  |  |  |  |  |  |  |  |  |  |  |  |  |  |  |  |  |
| 96F | Leopold |  | O |  |  |  |  |  |  |  |  |  |  |  |  |  |  |  |  |  |  |  |  |  |  |
| 103F | Druid |  | O | O | O | O | O |  |  |  |  |  |  |  |  |  |  |  |  |  |  |  |  |  |  |
| 104M | Druid |  | X |  |  |  |  |  |  |  |  |  |  |  |  |  |  |  |  |  |  |  |  |  |  |
| 105F | Druid |  |  |  | X | O | O | X | O |  |  |  |  |  |  |  |  |  |  |  |  |  |  |  |  |
| *106F* | *Druid* |  | X | X | X | X | O | O | O | O |  |  |  |  |  |  |  |  |  |  |  |  |  |  |  |
| *106F* | *Geode* |  |  |  |  |  |  |  |  |  |  |  | X | X |  |  |  |  |  |  |  |  |  |  |  |
| 113M | Agate |  |  |  |  |  |  |  |  |  |  |  |  |  |  |  |  |  |  |  | O |  |  |  |  |
| 148F | Leopold |  |  |  | X | X | X | O |  |  |  |  |  |  |  |  |  |  |  |  |  |  |  |  |  |
| *150M* | *Leopold* |  |  |  | O | O |  |  |  |  |  |  |  |  |  |  |  |  |  |  |  |  |  |  |  |
| *150M* | *Rose* |  |  |  |  |  |  | X | X | X | O |  |  |  |  |  |  |  |  |  |  |  |  |  |  |
| 151F | Leopold |  |  |  |  | O |  | X |  |  |  |  |  |  |  |  |  |  |  |  |  |  |  |  |  |
| 152F | Leopold |  |  |  | O |  |  |  |  |  |  |  |  |  |  |  |  |  |  |  |  |  |  |  |  |
| 153F | Rose |  |  |  | O |  |  |  |  |  |  |  |  |  |  |  |  |  |  |  |  |  |  |  |  |
| 154F | Rose |  |  |  | O |  |  |  |  |  |  |  |  |  |  |  |  |  |  |  |  |  |  |  |  |
| 155F | Rose |  |  |  | O | O | O |  |  |  |  |  |  |  |  |  |  |  |  |  |  |  |  |  |  |
| 156F | Rose |  |  |  |  |  | O |  |  |  |  |  |  |  |  |  |  |  |  |  |  |  |  |  |  |
| 161M | Rose |  |  |  | X |  |  |  |  |  |  |  |  |  |  |  |  |  |  |  |  |  |  |  |  |
| 162M | Rose |  |  |  |  |  | O |  |  |  |  |  |  |  |  |  |  |  |  |  |  |  |  |  |  |
| 163M | Druid |  |  |  | O |  |  |  |  |  |  |  |  |  |  |  |  |  |  |  |  |  |  |  |  |
| 190F | Rose |  |  |  |  |  | X | X | O | O | O |  |  |  |  |  |  |  |  |  |  |  |  |  |  |
| 207M | Rose |  |  |  |  |  |  |  | X | X | X |  |  |  |  |  |  |  |  |  |  |  |  |  |  |
| 209F | Leopold |  |  |  |  |  |  |  | O | O |  |  |  | X | O |  | O | X | X |  | O | X |  |  |  |
| 210M | Leopold |  |  |  |  |  |  |  | O | O |  |  |  |  |  |  |  |  |  |  |  |  |  |  |  |
| 211M | Leopold |  |  |  |  |  |  |  | O | X |  |  |  |  |  |  |  |  |  |  |  |  |  |  |  |
| 216F | Druid |  |  |  |  |  |  |  | X | O |  |  |  |  |  |  |  |  |  |  |  |  |  |  |  |
| 217F | Druid |  |  |  |  |  |  |  | O | O |  |  |  |  |  |  |  |  |  |  |  |  |  |  |  |
| 218F | Druid |  |  |  |  |  |  |  | O | O |  |  |  |  |  |  |  |  |  |  |  |  |  |  |  |
| 219M | Druid |  |  |  |  |  |  |  | O |  |  |  |  |  |  |  |  |  |  |  |  |  |  |  |  |
| 220F | Leopold |  |  |  |  |  |  |  | X | O | X | X | O |  |  |  |  |  |  |  |  |  |  |  |  |
| 222M | Druid |  |  |  |  |  |  |  | X |  |  |  |  |  |  |  |  |  |  |  |  |  |  |  |  |
| 223F | Druid |  |  |  |  |  |  |  | O |  |  |  |  |  |  |  |  |  |  |  |  |  |  |  |  |
| 224M | Druid |  |  |  |  |  |  |  | O | X |  |  |  |  |  |  |  |  |  |  |  |  |  |  |  |
| 227M | Geode |  |  |  |  |  |  |  |  |  |  |  |  |  |  |  | X |  |  |  |  |  |  |  |  |
| 253M | Druid |  |  |  |  |  |  |  |  |  | O |  | O | X | O |  |  |  |  |  |  |  |  |  |  |
| 254M | Druid |  |  |  |  |  |  |  |  |  | X |  |  |  |  |  |  |  |  |  |  |  |  |  |  |
| 255F | Druid |  |  |  |  |  |  |  |  |  | O | X | O | X |  | O |  |  |  |  |  |  |  |  |  |
| 259F | Leopold |  |  |  |  |  |  |  |  |  | O | X | O |  |  |  |  |  |  |  |  |  |  |  |  |
| 260F | Rose |  |  |  |  |  |  |  |  |  | X |  |  |  |  |  |  |  |  |  |  |  |  |  |  |
| 286F | Druid |  |  |  |  |  |  |  |  |  |  |  | O |  |  |  |  |  |  |  |  |  |  |  |  |
| *287M* | *Leopold* |  |  |  |  |  |  |  |  |  |  |  | X | O | X | X | O |  |  |  |  |  |  |  |  |
| *287M* | *Hellroaring* |  |  |  |  |  |  |  |  |  |  |  |  |  |  |  |  | X | X | X |  |  |  |  |  |
| 288F | Leopold |  |  |  |  |  |  |  |  |  |  |  | X | O | O |  |  |  |  |  |  |  |  |  |  |
| 289M | Leopold |  |  |  |  |  |  |  |  |  |  |  | O | O |  |  |  |  |  |  |  |  |  |  |  |
| 290F | Leopold |  |  |  |  |  |  |  |  |  |  |  | O | X |  |  |  |  |  |  |  |  |  |  |  |
| 294M | Geode |  |  |  |  |  |  |  |  |  |  |  | O |  |  |  |  |  |  |  |  |  |  |  |  |
| 300M | Geode |  |  |  |  |  |  |  |  |  |  |  | X |  |  |  |  |  |  |  |  |  |  |  |  |
| *302M* | *Druid* |  |  |  |  |  |  |  |  |  |  |  |  |  |  |  |  |  |  |  | O | X | X |  |  |
| *302M* | *Blacktail* |  |  |  |  |  |  |  |  |  |  |  |  |  |  |  |  |  |  |  |  |  |  | X | X |
| 344F | Leopold |  |  |  |  |  |  |  |  |  |  |  |  | O | O |  |  |  |  |  |  |  |  |  |  |
| 345F | Leopold |  |  |  |  |  |  |  |  |  |  |  |  | O | X | X | X | X | X | O |  |  |  |  |  |
| 348M | Druid |  |  |  |  |  |  |  |  |  |  |  |  |  | O | X |  |  |  |  |  |  |  |  |  |
| 349M | Druid |  |  |  |  |  |  |  |  |  |  |  |  |  | X |  |  |  |  |  |  |  |  |  |  |
| 350M | Druid |  |  |  |  |  |  |  |  |  |  |  |  |  | X |  |  |  |  |  |  |  |  |  |  |
| 351M | Geode |  |  |  |  |  |  |  |  |  |  |  |  | X |  |  |  |  |  |  |  |  |  |  |  |
| 352M | Geode |  |  |  |  |  |  |  |  |  |  |  |  | O |  |  |  |  |  |  |  |  |  |  |  |
| 353F | Geode |  |  |  |  |  |  |  |  |  |  |  |  | O | O |  |  |  |  |  |  |  |  |  |  |
| 353F | Hellroaring |  |  |  |  |  |  |  |  |  |  |  |  |  |  |  |  |  | X | O |  |  |  |  |  |
| *374M* | *Druid* |  |  |  |  |  |  |  |  |  |  |  |  |  | O |  |  |  |  |  |  |  |  |  |  |
| *374M* | *Geode* |  |  |  |  |  |  |  |  |  |  |  |  |  |  | X | O |  |  |  |  |  |  |  |  |
| 375F | Druid |  |  |  |  |  |  |  |  |  |  |  |  |  | O | X |  |  |  |  |  |  |  |  |  |
| 376F | Druid |  |  |  |  |  |  |  |  |  |  |  |  |  | O |  |  |  |  |  |  |  |  |  |  |
| 377M | Slough |  |  |  |  |  |  |  |  |  |  |  |  |  |  |  | O | X | O |  |  |  |  |  |  |
| 380F | Slough |  |  |  |  |  |  |  |  |  |  |  |  |  |  |  |  |  | O | X |  |  |  |  |  |
| 381M | Leopold |  |  |  |  |  |  |  |  |  |  |  |  |  | O | O | O |  |  |  |  |  |  |  |  |
| 391F | Geode |  |  |  |  |  |  |  |  |  |  |  |  |  | X | X | O |  |  |  |  |  |  |  |  |
| 392M | Geode |  |  |  |  |  |  |  |  |  |  |  |  |  | X |  |  |  |  |  |  |  |  |  |  |
| 453M | Slough |  |  |  |  |  |  |  |  |  |  |  |  |  |  |  | X |  |  |  |  |  |  |  |  |
| *468M* | *Leopold* |  |  |  |  |  |  |  |  |  |  |  |  |  |  |  | X |  |  |  |  |  |  |  |  |
| *468M* | *Hellroaring* |  |  |  |  |  |  |  |  |  |  |  |  |  |  |  |  | X | O | X |  |  |  |  |  |
| 469F | Leopold |  |  |  |  |  |  |  |  |  |  |  |  |  |  |  | O | O |  | X | O |  |  |  |  |
| *470F* | *Leopold* |  |  |  |  |  |  |  |  |  |  |  |  |  |  |  | O | O |  |  |  |  |  |  |  |
| *470F* | *Oxbow* |  |  |  |  |  |  |  |  |  |  |  |  |  |  |  |  |  |  |  |  | X |  |  |  |
| 471F | Agate |  |  |  |  |  |  |  |  |  |  |  |  |  |  |  |  |  |  |  | X |  |  |  |  |
| 480M | Druid |  |  |  |  |  |  |  |  |  |  |  |  |  |  |  |  |  |  |  | O | O | O | X | O |
| 483F | Geode |  |  |  |  |  |  |  |  |  |  |  |  |  |  |  | X |  |  |  |  |  |  |  |  |
| 488M | Geode |  |  |  |  |  |  |  |  |  |  |  |  |  |  |  | O |  |  |  |  |  |  |  |  |
| 489M | Slough |  |  |  |  |  |  |  |  |  |  |  |  |  |  |  | O |  | X |  |  |  |  |  |  |
| 490M | Slough |  |  |  |  |  |  |  |  |  |  |  |  |  |  |  | X | O | O | O |  |  |  |  |  |
| 491M | Slough |  |  |  |  |  |  |  |  |  |  |  |  |  |  |  | O | X |  |  |  |  |  |  |  |
| 524F | Agate |  |  |  |  |  |  |  |  |  |  |  |  |  |  |  |  |  |  |  | O |  |  |  |  |
| 525F | Agate |  |  |  |  |  |  |  |  |  |  |  |  |  |  |  |  |  |  |  | X |  |  |  |  |
| 526F | Slough |  |  |  |  |  |  |  |  |  |  |  |  |  |  |  |  |  | O | X |  |  |  |  |  |
| 527F | Slough |  |  |  |  |  |  |  |  |  |  |  |  |  |  |  |  |  | X |  |  |  |  |  |  |
| 534M | Leopold |  |  |  |  |  |  |  |  |  |  |  |  |  |  |  |  |  | O | X | X | O | O |  |  |
| 536F | Oxbow |  |  |  |  |  |  |  |  |  |  |  |  |  |  |  |  |  |  |  |  | X | X |  |  |
| 569F | Druid |  |  |  |  |  |  |  |  |  |  |  |  |  |  |  |  |  |  |  | X | O | X | O | X |
| 571F | Druid |  |  |  |  |  |  |  |  |  |  |  |  |  |  |  |  |  |  |  | X | X | O |  |  |
| 588F | Leopold |  |  |  |  |  |  |  |  |  |  |  |  |  |  |  |  |  |  |  |  | X |  |  |  |
| 590M | Agate |  |  |  |  |  |  |  |  |  |  |  |  |  |  |  |  |  |  |  | O |  |  |  |  |
| 591F | Leopold |  |  |  |  |  |  |  |  |  |  |  |  |  |  |  |  |  |  |  | X | O |  |  |  |
| 592F | Leopold |  |  |  |  |  |  |  |  |  |  |  |  |  |  |  |  |  |  |  | O |  |  |  |  |
| 593F | Leopold |  |  |  |  |  |  |  |  |  |  |  |  |  |  |  |  |  |  |  | O | O |  |  |  |
| 623F | Leopold |  |  |  |  |  |  |  |  |  |  |  |  |  |  |  |  |  |  |  |  |  | X |  |  |
| 624F | Leopold |  |  |  |  |  |  |  |  |  |  |  |  |  |  |  |  |  |  |  |  |  | O |  |  |
| 625F | Leopold |  |  |  |  |  |  |  |  |  |  |  |  |  |  |  |  |  |  |  |  |  | X |  |  |
| 626F | Oxbow |  |  |  |  |  |  |  |  |  |  |  |  |  |  |  |  |  |  |  |  |  | O |  |  |
| 627M | Oxbow |  |  |  |  |  |  |  |  |  |  |  |  |  |  |  |  |  |  |  |  |  | O |  |  |
| 628M | Oxbow |  |  |  |  |  |  |  |  |  |  |  |  |  |  |  |  |  |  |  |  |  | X |  |  |
| 642F | Blacktail |  |  |  |  |  |  |  |  |  |  |  |  |  |  |  |  |  |  |  |  |  |  | X | O |
| 645F | Druid |  |  |  |  |  |  |  |  |  |  |  |  |  |  |  |  |  |  |  |  |  | O | X | X |
| 684M | Everts |  |  |  |  |  |  |  |  |  |  |  |  |  |  |  |  |  |  |  |  |  |  |  | X |
| 685M | Everts |  |  |  |  |  |  |  |  |  |  |  |  |  |  |  |  |  |  |  |  |  |  |  | X |
| 690F | Druid |  |  |  |  |  |  |  |  |  |  |  |  |  |  |  |  |  |  |  |  |  |  |  | O |
| 691F | Druid |  |  |  |  |  |  |  |  |  |  |  |  |  |  |  |  |  |  |  |  |  |  |  | O |
| 693F | Blacktail |  |  |  |  |  |  |  |  |  |  |  |  |  |  |  |  |  |  |  |  |  |  |  | X |
