# Supplementary material for: Effect of Sociality and Season on Gray Wolf (Canis lupus) Foraging Behavior: Implications for Estimating Summer Kill Rate
Source: PLoS One. 2011 Mar 1;6(3):e17332. doi: 10.1371/journal.pone.0017332 (PMC3046980; doi:10.1371/journal.pone.0017332)
Supplement: Text S1 — Description of the GPS collar used during summer 2004. (DOC) [file pone.0017332.s005.doc]

# Text S1. Description of the GPS collar used during summer 2004.

We programmed the first GPS collar that we used (in 2004) to record 40 locations per day from 1 May – 31 July. Specifically, it recorded one location every 60 minutes during eight hours of each day (0600-1400), and recorded a location every 30 minutes for the remainder of the day. Due to this schedule, we likely did not detect carcasses attended by this wolf that would have been detected if the collar had collected a location every 30 minutes. To correct the number of carcasses detected in 2004, we used the data from 2008 and 2009 as reference. See *GPS Collar Success Simulations* for additional information about why this data was used. The reference data provided instances of individual wolf detection at 183 large ungulate carcasses and 174 small ungulate carcasses. We simply removed eight locations per day from the reference data and examined the number of carcasses no longer detected (i.e. carcasses which no longer met the spatial and temporal requirements for individual wolf detection). Specifically, we removed daily locations collected at 0630, 0730, 0830, 0930, 1030, 1130, 1230, and 1330 to replicate the 2004 GPS collar fix schedule. After removing these locations, 92.4% of large ungulates and 85.6% of small ungulates were still detected in 2008 and 2009. Therefore, prior to applying Eq. 3 to the 2004 data, we corrected the number of carcasses detected by dividing the number of large ungulate carcasses (*n* = 9) by 0.924 and the number of small ungulate carcasses (*n* = 5) by 0.856.
