# Supplementary material for: Effect of Sociality and Season on Gray Wolf (Canis lupus) Foraging Behavior: Implications for Estimating Summer Kill Rate
Source: PLoS One. 2011 Mar 1;6(3):e17332. doi: 10.1371/journal.pone.0017332 (PMC3046980; doi:10.1371/journal.pone.0017332)
Supplement: Text S2 — Investigation of single, isolated GPS locations. (DOC) [file pone.0017332.s006.doc]

# Text S2. Investigation of single, isolated GPS locations.

In 2008, 19.5% of all GPS locations were not associated with any cluster, but were single, isolated locations. To assess the possibility of undiscovered prey in our study system, we searched 1045 of the single locations in 2008. Specifically, we randomly selected 40% of the single locations and searched as many of these as time permitted (i.e., we searched 77% of the randomly selected sites). We rarely found any sign of prey remains at single locations. More specifically, we found ungulate carcasses from the previous winter (which provided no significant biomass) at 32 of the 1045 single locations we searched. At nine of the 1045 locations, we found bone shards of neonate ungulates. At least some of these nine locations likely represented locations where wolves did not obtain biomass. That is, wolves often visit carcasses whose edible biomass had been previously consumed [13]. Because this kind of behavior is consistent with spending less than 30 minutes (the time between two consecutive GPS locations), we did not include the neonate ungulates found at single locations. We also found small prey, such as blue grouse, at 2 of the 1045 single locations. Because we rarely found any sign of prey remains in 2008, we did not search single locations in subsequent years.
